# Clinical characteristics and outcomes of patients with TSH-secreting pituitary adenoma and Graves’ disease - a case report and systematic review

**DOI:** 10.1186/s13044-023-00184-2

**Published:** 2024-02-05

**Authors:** Adeel Ahmad Khan, Shahd I. Ibrahim, Fateen Ata, Bara Wazwaz, Mohammad Abdulalim Hanoun, Sirajeddin Belkhair, Zaina Seros Rohani, Zeinab Dabbous

**Affiliations:** 1 Department of Endocrinology, Hamad Medical Corporation, Doha, Qatar; 2 Department of Pathology, Hamad Medical Corporation, Doha, Qatar; 3 Department of Radiology, Hamad Medical Corporation, Doha, Qatar; 4 Department of Neurosurgery, Hamad Medical Corporation, Doha, Qatar

**Keywords:** Hyperthyroidism, TSH-secreting pituitary adenoma, Graves’ disease

## Abstract

**Background:**

Coexistence of TSH-secreting pituitary adenoma (TSHoma) and Graves’ disease (GD) is rare and complicates the management decision.

**Methods:**

We present a case of the co-existence of TSHoma and GD. In addition, we systematically searched articles describing TSHoma and GD in the same patient published until 20th March 2023, using Pubmed, Scopus and Embase.

**Case presentation:**

A 46-year-old man presented with symptoms of thyrotoxicosis. His thyroid function tests showed serum TSH 3.35 (reference range 0.3–4.2) mIU/L, FT3 19.7 (3.7–6.4) pmol/L, and FT4 68.9 (11-23.3) pmol/L. The serum TSH receptor antibody was 11.5 mIU/L (positive at ≥ 1.75 mIU/L). Pituitary magnetic resonance imaging showed macroadenoma compressing the optic chiasm. The patient underwent trans-sphenoidal resection of pituitary adenoma. Postoperatively, he remained on maintenance carbimazole and octreotide.

**Results:**

Fourteen articles comprising 15 patients were identified from the systemic search. A total of 16 patients (including the current case) were included in the systematic review. The mean (± SD) age at diagnosis was 41 ± 13.6 years. The majority were females (75%). The median (IQR) TSH was 1.95 (0.12–5.5) mIU/L, the median (IQR) free T3 was 11.7 (7.6–19.7) pmol/L and the median (IQR) free T4 level was 47.6 (33.3–64.4) pmol/L. Ten (76.9%) patients had positive TSH receptor antibody levels. 84.6% had pituitary macroadenoma. Pituitary surgery was performed in 12 (75%) patients. At the last follow-up, 4 (25%) patients had complete resolution of symptoms after pituitary surgery, 3 (18.7%) were on maintenance treatment with thionamides for GD, 1 (6.25%) on beta-blockers and 1 (6.25%) on somatostatin analog.

**Conclusion:**

TSHoma and GD can co-exist, and it is essential to identify this rare association as it can significantly impact treatment strategies.

## Introduction

TSH-secreting pituitary adenoma (TSHoma) is a rare cause of hyperthyroidism and results from abnormal clonal expansion of the TSH-producing pituitary cells. The prevalence of TSHoma is only up to 0.94% of all pituitary adenomas [1]. As per a study based on the Swedish Pituitary Registry, the incidence of TSHoma is 0.15 per million population per year and a prevalence of 2.8 per million [2]. There is also an increased rate of diagnosis of TSHoma, with the incidence increasing from 0.05 per million per year to 0.26 per million per year from 1990 to 2009 [3]. The disease is usually seen in middle-aged patients. However, the literature reports an age range of 11–84 years at diagnosis [1]. The diagnosis is generally suspected when the syndrome of inappropriate thyroid stimulating hormone (TSH) secretion (SITSH) is noted due to unsuppressed TSH levels in the presence of high thyroid hormone levels [4]. However, it is essential to rule out resistance t thyroid hormone laboratory assay interference, and mutations in T4-binding globulins (TBG) [5]. Thyrotropin-releasing hormone (TRH) stimulation test is an important tool to identify TSHoma as a probable etiology of SITSH, with literature reporting an abnormal response of TSH to TRH stimulation in up to 90% of cases. Lack of inhibition of TSH after T3 administration (T3 suppression test) and high levels of serum glycoprotein hormone alpha-subunit (α-GSU) also indicate TSHoma as the most likely etiology of SITSH [6]. Pituitary MRI, radio-labeled somatostatin scintigraphy, and positron emission tomography (PET) scan are the imaging modalities used to diagnose TSHoma [1]. First-line treatment involves surgical resection of the adenoma. In patients who are not cured with surgical management, medical treatment with somatostatin analogs and radiotherapy are available options [6].

Graves’ disease (GD) is one of the most common causes of hyperthyroidism, with a reported incidence rate of around 25 per 100,000 population [6]. The condition is more common in females, with the female-to-male ratio as high as 5.6:1. TSH receptor antibodies (TRAb) have over 97% sensitivity in diagnosing GD [7]. Co-occurrence of GD and TSHoma in the same patient is rare and complicates the clinical picture [8–10]. It is essential to identify the co-existence of TSHoma and GD simultaneously in a patient and to accurately differentiate GD or TSHoma as a potential etiology of hyperthyroidism in a patient with a history of either condition, as the treatment of these conditions differs. Anti-thyroid drugs are the drugs of choice to achieve a euthyroid state in a patient with GD. However, in patients with TSHoma, the use of anti-thyroid drugs can potentially lead to an increase in TSH level due to inhibition of negative feedback, thus leading to worsening and an increase in the size of TSHoma [8]. Due to its rarity, there is little knowledge regarding managing patients where both diagnoses are detected. In this article, we present a case of a 46-year-old gentleman with co-existing TSHoma and GD. In addition, we systematically reviewed all the available published articles reporting the existence of TSHoma and GD in the same patient.

## Materials and methods

The protocol for the systematic review has been registered in the International Prospective Register of Systematic Reviews (PROSPERO) with the protocol ID CRD42023408008. The systematic review was conducted according to the Preferred Reporting Items for Systematic Reviews and Meta-Analyses (PRISMA) guidelines [11].

### Literature search

We conducted a literature search of all the articles published until 20th March 2023. Electronic databases including Pubmed, Scopus, and Embase were used to search eligible articles for the review using the following search terms: “TSHoma,“ “TSH secreting adenoma,“ “TSH secreting tumor,“ “Thyrotropin secreting tumor,“ “pituitary adenoma,“ “pituitary tumor,“ “Thyrotropinoma,“ “Thyrotropin secreting adenoma,“ “Graves’ disease,“ “Basedow disease,“ “Graves’ hyperthyroidism,“ “Graves’ thyrotoxicosis.“

### Study selection

**Fig. 1:**
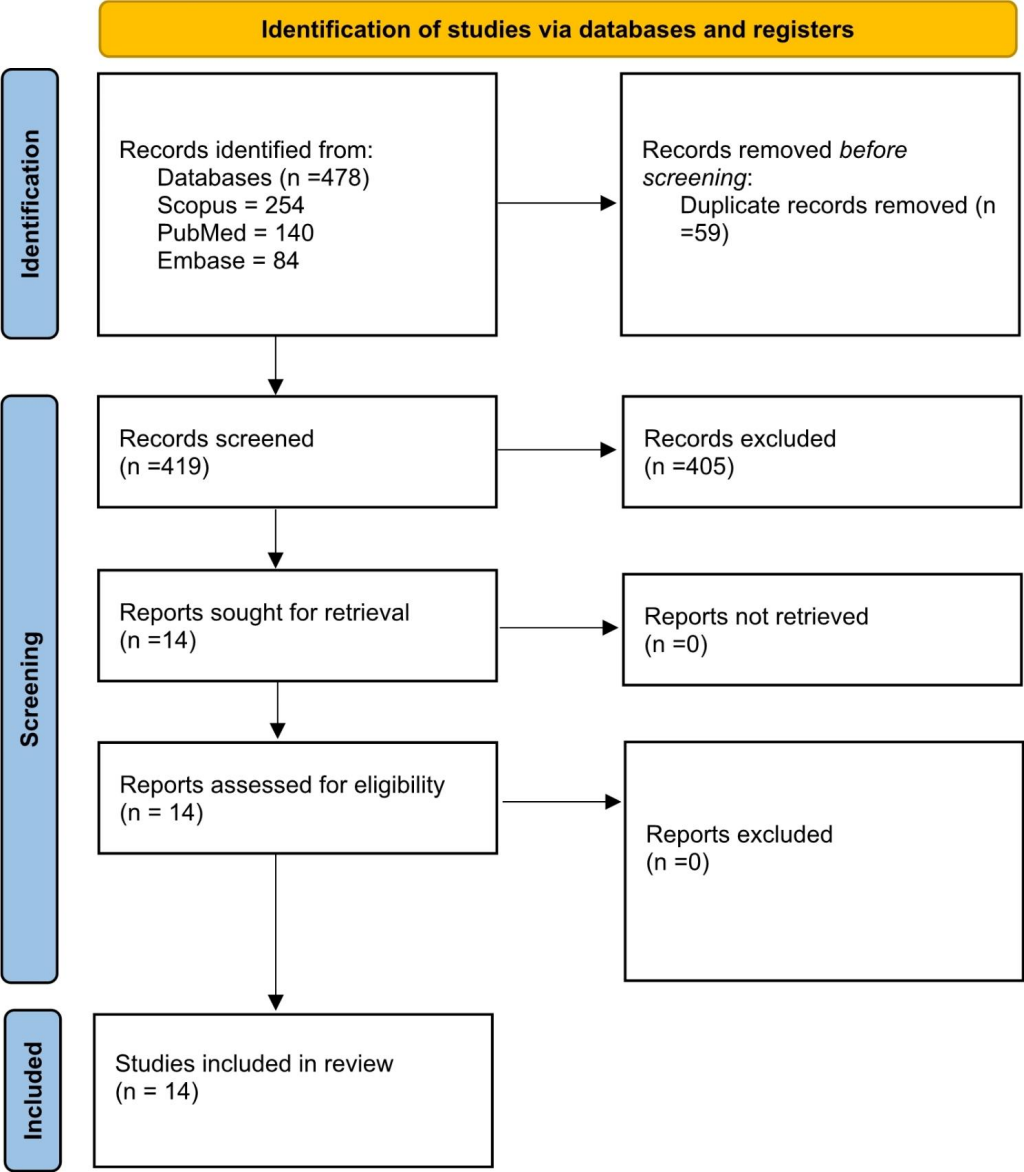
PRISMA flowsheet of the systematic review article selection process

All the retrieved articles were screened initially by the title and abstract by two members (SI and FA). Any disagreement found during the initial screening process was sorted by a third reviewer (AAK) through the independent review of the articles. The full-text review was subsequently performed if the article was considered eligible for inclusion in the systematic review based on initial screening (Fig. 1).

### Inclusion criteria

Case reports, case series and original research articles describing TSHoma and GD in the same patient and published in English from any date till 20th March 2023 were included in the study.

### Exclusion criteria

Articles published in languages other than English, conference abstracts with insufficient data and reports only on TSHoma or only on GD were excluded.

### Quality assessment

SI and FA did the quality assessment of the included articles. Case reports and series were assessed by Joanna Briggs Institute (JBI) case report appraisal checklist for inclusion in systematic reviews [12]. AAK resolved disagreements in the quality assessment process.

### Data collection and statistical analysis

The study members extracted data on patient demographics, clinical presentations, laboratory and imaging investigations, management, and clinical outcomes. Descriptive statistics were performed to describe the results of the systematic review. We used mean (SD) and median (IQR) to report continuous variables and frequency to describe the categorical variables. We used STATA 17 to conduct the statistical analysis for the study.

## Results

### Case presentation

A 46-year-old Filipino gentleman presented to the emergency department with a decreased vision for one month. He also reported three months history of tremors and palpitations. There was no history of headache, weakness, numbness, altered level of consciousness, unintentional weight loss, chronic diarrhea, insomnia, shortness of breath, neck swelling or pain. The patient was diagnosed with hypertension five months ago and took atenolol 50 mg once daily for management. There was no family history of thyroid or autoimmune diseases. The patient did not smoke or drink alcohol.

On physical examination, the patient had high blood pressure (176/86) with a normal heart rate, respiratory rate, temperature and oxygen saturation. He had mild bilateral hand tremors. Neurological examination revealed bitemporal hemianopia. He had a firm, diffuse, non-tender thyroid swelling. There was no proptosis, lid retraction, lid lag, muscle weakness, thyroid bruit, or skin changes. The patient had a pale left-sided optic disc on the fundus examination. The cardiac, respiratory and gastrointestinal examination was normal.

Laboratory examination showed a normal complete blood count and renal and liver function tests. Serum TSH was 3.35 (reference range 0.3–4.2) mIU/L, FT3 was 19.7 (3.7–6.4) pmol/L, and FT4 was 68.9 (11-23.3) pmol/L. Repeated thyroid function tests (TFT) showed similar results. The serum TSH receptor antibody was positive (11.5 mIU/L; Cut-off ≥ 1.75 mIU/L). Serum sex hormone binding globulin (SHBG) was high (126 nmol/L; reference range: 18.3–54.1 nmol/L). The rest of the pituitary hormonal profile was normal.

**Fig. 2:**
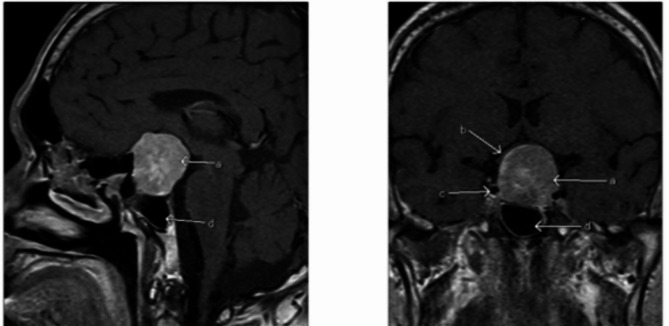
Sagittal and coronal views of Magnetic Resonance Imaging (MRI) of the pituitary gland at the initial presentation showing large enhancing sellar and suprasellar mass lesion (**a**), compressed and elevated optic chiasm (**b**), and tumor contacting cavernous portion of the internal carotid artery (**c**) and sphenoid sinus (**d**)

A thyroid ultrasound showed enlarged inhomogeneous echogenicity and increased vascularity, indicating diffuse thyroid disease. MRI of the pituitary gland showed a large sellar/suprasellar mass of intermediate T1 signal and a mixture of hypo and hyperintense signal in T2 with moderate enhancement. Optic chiasm was elevated and bowed over the superior border. The anterior carotid artery was displaced superiorly with no encasement, and the tumor was contacting the left cavernous internal carotid artery (ICA) (Fig. 2).

**Fig. 3:**
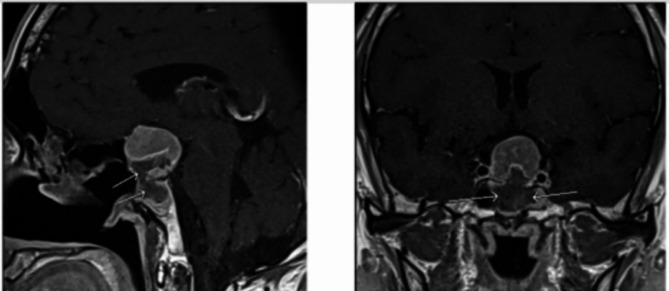
Post-operative Magnetic Resonance Imaging (MRI) of the pituitary (post-contrast) gland showing debulking of the tumor (arrows)

The patient was diagnosed with TSHoma with possibly co-existing Grave’s disease (due to the presence of positive TSH receptor antibody and diffuse thyroid enlargement). Octreotide, subcutaneous 100 mcg twice a day, was started. Repeat TFTs showed a decrease in TSH level to 0.45 mIU/L (> 50% reduction) and a decrease in FT4 to 50.9 pmol/L. At this point, carbimazole 20 mg twice daily was added, further decreasing FT4 to 39.9 pmol/L. Due to a lack of availability, the TRH stimulation test was not performed. The patient underwent navigation-assisted trans-nasal, trans-sphenoidal endoscopic resection of pituitary macroadenoma. Repeat MRI pituitary 48 h post-operation showed a solid residual enhancement measuring 28 × 24.5 × 19.5 mm (Fig. 3**).**

**Fig. 4:**
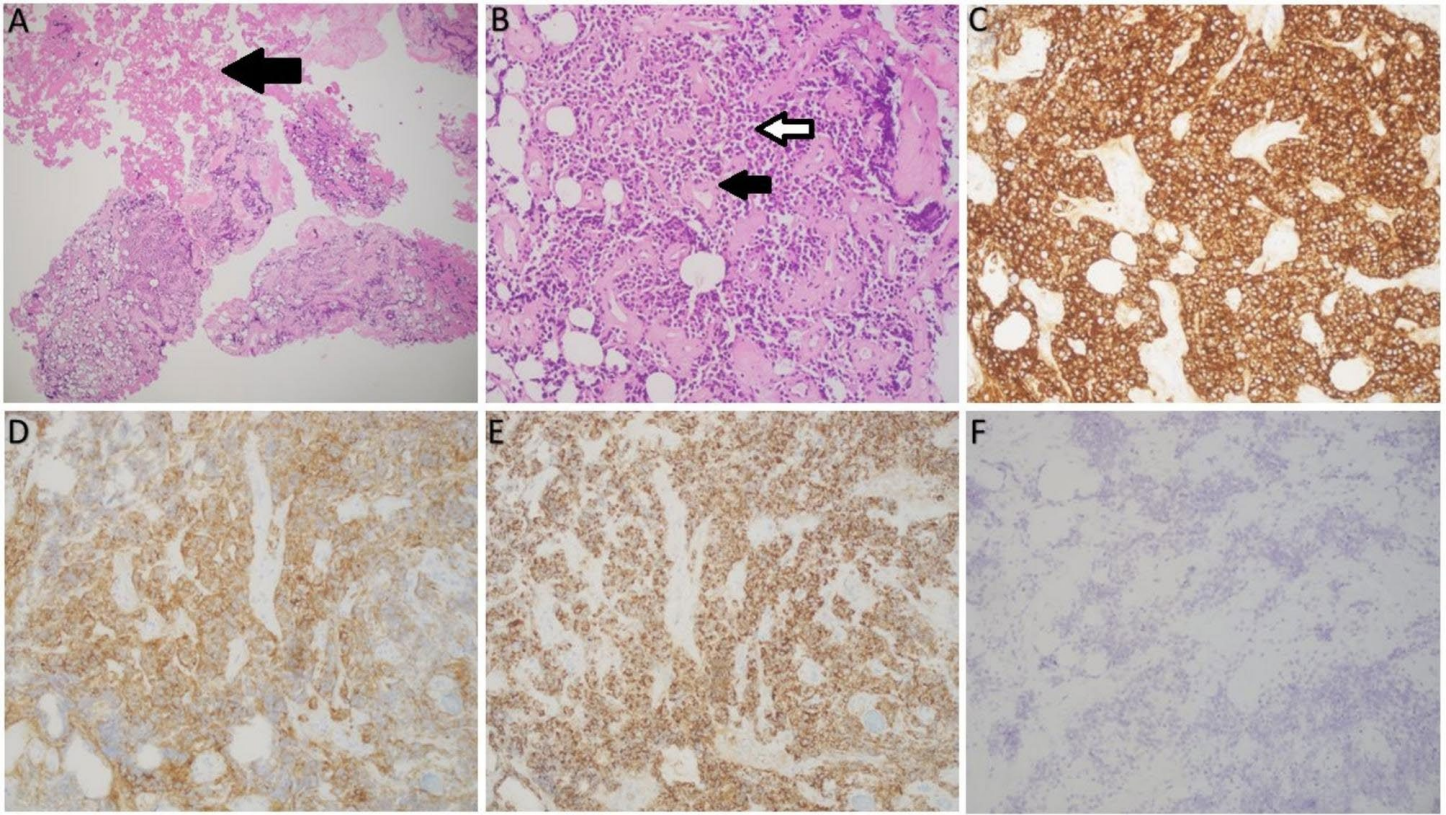
Histopathology and immunohistochemistry of the resected pituitary tissue specimen. Sections show fragments of neuroendocrine tumor in a background of abundant fibrin deposition and hyalinized small blood vessels (black arrow fibrin) (**A**). The tumor cells are monomorphic with round nuclei and vesicular chromatin. No necrosis or mitotic figures seen (Black arrow hyalinized blood vessel, white arrow tumor cells) (**B**). By immunohistochemistry, tumor cells are strongly positive for synaptophysin (**C**), chromogranin (**D**) and cytokeratin CK AE1/AE3 (**E**) confirming the neuroendocrine origin of the neoplasm. Meanwhile, all other hormonal markers including TSH are negative (**F**)

The histopathology revealed marked perivascular and interstitial fibrosis. Immunohistochemistry (IHC) staining was negative for Adrenocorticotrophic hormone (ACTH), Prolactin, Growth Hormone (GH), Follicle Stimulating Hormone (FSH), Thyroid Stimulating Hormone (TSH) and Luteinizing Hormone (LH), and positive for synaptophysin. (Fig. 4).

**Fig. 5:**
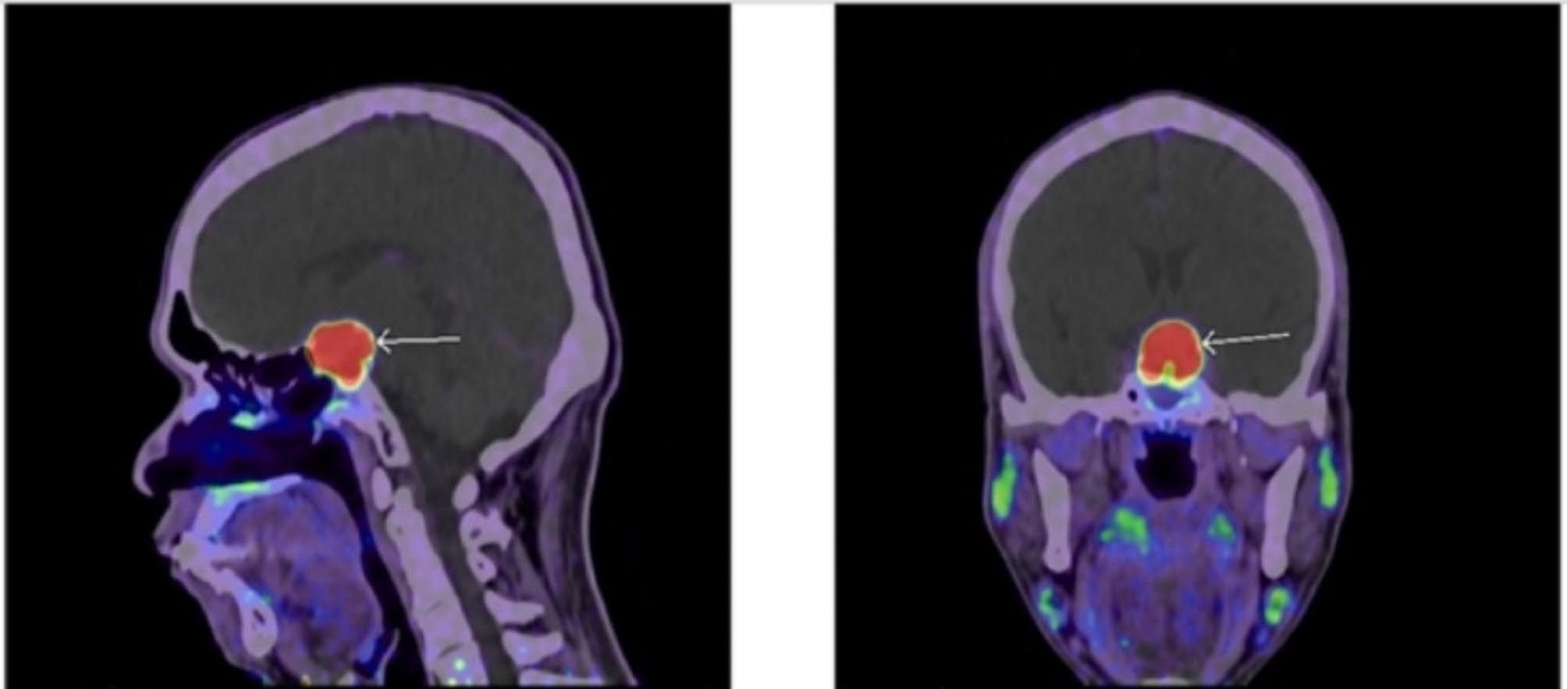
Sagittal and coronal views of 68-Gallium DOTA peptide Positron Emission Tomography (Ga68 DOTATATE PET CT) of the pituitary gland with the arrow showing significant uptake corresponding to pituitary macroadenoma

Given the unexpected histopathological finding, the alpha subunit pituitary tumor marker level was sent and was positive (0.6 ng/ml, cut off for normal is </= 0.5 ng/ml), supporting the diagnosis of TSH secreting pituitary adenoma. An NM Ga68 DOTATATE whole-body PET CT (somatostatin receptor scintigraphy) showed significant uptake in the pituitary macroadenoma (Fig. 5). A positiveGa68 DOTATATE is suggestive of functioning pituitary tissue in the adenoma [13].

The patient had a nuclear scan of the thyroid gland (192 MBq Tc 99 m pertechnetate), demonstrating features suggestive of GD (diffusely increased homogeneous uptake and thyroid uptake was 36%).

**Fig. 6:**
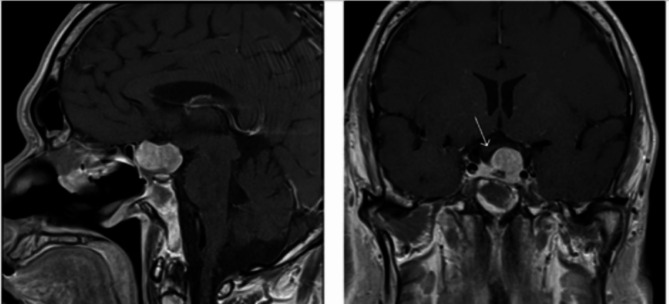
Follow-up Magnetic Resonance Imaging (MRI) of pituitary gland 1-year post operatively showing regression in lesion size. The optic chiasm is decompressed (arrow)

On follow-up, the patient had persistent visual field defect but noticed much improvement in right eye vision and mild improvement in left eye vision. A follow-up pituitary MRI showed a stable residual tumor size. The optic chiasma was still compressed and elevated more on the left side. The patient was offered a second surgery, but he refused and opted for medical management. He is now on octreotide intramuscular injection of 20 mg every four weeks and carbimazole 10 mg once daily with the latest TFTs in the normal range. His latest MRI pituitary performed one year after the surgery shows regression in the size of the residual pituitary adenoma measuring 24 × 22 × 19 mm (Fig. 6).

### Systematic review results

Fourteen case reports fulfilled the eligibility criteria [8–10, 14–24]. Table 1 summarizes the clinical details of the added cases.

**Table 1:** Clinical characteristics and outcomes of patients included in the study

Authors	Year	Age	Gender	Clinical Features	Diagnosis	Antibodies detected	Micro/macro	Final outcome
Arai N et al. [8]	2017	40	F	Exophthalmos	Simultaneous GD and TSHomaat diagnosis of HTH	TRAB, Anti TPO AB, Anti-TG AB	Macroadenoma	Resolution after surgery
K. Kageyama [16]	2007	21	F	Palpitations, weight loss, goiter	TSHoma first followed by GD	TRAB	Macroadenoma	Resolution after surgery
Kamoun et al. [9]	2014	36	F	Palpitations, heat intolerance, tremors, exophthalmos, goiter	Simultaneous GD and TSHoma at diagnosis of HTH	TRAB, Anti TPO AB	Macroadenoma	Resolution after surgery
Koriyama et al. [18]	2004	31	F	Weight loss, sweating, goiter	TSHoma first followed by GD	Thyroid stimulation AB, anti-TPO AB, anti-TG AB	Macroadenoma	On treatment for GD
Lee et al. [19]	2010	27	M	Palpitations, goiter	GD diagnosed first followed by TSHoma development	TRAB	Macroadenoma	Not mentioned
Lee et al. [19]	2010	28	F	Palpitations, tremors, goiter	GD diagnosed first followed by TSHoma development	TRAB	Macroadenoma	Not mentioned
Li et al. [10]	2018	55	M	Atrial fibrillation, heat intolerance, tremors, visual field defect	Simultaneous GD and TSHoma at diagnosis of hyperthyroidism	TRAB	Macroadenoma	Post op, he still has a residual mass with normal TSH.
Ogawa et al. [21]	2013	32	F	Palpitations, weight loss	GD diagnosed first followed by TSHoma development	TRAB	Microadenoma	Resolution after surgery
Okuyucu et al. [22]	2016	37	F	Exophthalmos	Simultaneous GD and TSHoma at diagnosis of hyperthyroidism	TRAB	Macroadenoma	Not mentioned
Quinn et al. [23]	2020	68	F	Asymptomatic	GD diagnosed first followed by TSHoma development		Microadenoma	Euthyroid on BB
Sandler [24]	1976	53	F	Acromegaly (pre-existing), exophthalmos	Simultaneous GD and TSHoma at diagnosis of hyperthyroidism		Not reported	On treatment for GD
Donnell et al. [20]	1968	25	M	Heat intolerance, weight loss, sweating, tremors, visual field defect	TSHoma first followed by GD		Not reported	Not mentioned
Kamoi et al. [17]	1985	46	F	Heat intolerance, weight loss, sweating, tremors, goiter	TSHoma first followed by GD	Anti-TG AB	Not reported	Not mentioned
Fu et al. [15]	2020	55	F	Palpitations, heat intolerance, weight loss, sweating, tremors	Simultaneous GD and TSHoma at diagnosis of hyperthyroidism	TRAB, thyroid stimulation AB, anti-TG AB	Macroadenoma	On treatment for GD
Diri et al. [14]	2016	56	F	Palpitations, heat intolerance, headache	Simultaneous GD and TSHoma at diagnosis of hyperthyroidism	Anti TPO AB	Macroadenoma	Not mentioned
Present case	NA	46	M	Palpitations, tremors, goiter, visual field defect	Simultaneous GD and TSHoma at diagnosis of hyperthyroidism	TRAB	Macroadenoma	Maintained on octreotide

TSHOMA: Thyroid stimulating hormone secreting pituitary adenoma; GD: Graves’ disease; TPO: Thyroid Peroxidase; AB; Antibody; TRAB: Thyroid Stimulating Hormone Receptor Antibody: TG: Thyroglobulin; BB: Beta-blocker; F: Female; M: Male

Table 2 summarizes the demographic, clinical/laboratory characteristics, and outcomes of patients with TSHoma and GD. A total of 16 patients (including the current case) were included in the systematic review.

**Table 2:** Demographic, clinical/laboratory characteristics, and outcomes of patients with TSHoma and GD

Variable	Result
Number of patients (N)	16
Age in years (Mean +/- SD)	41 +/- 13.6
**Gender, N (%)**	
Male	4 (25)
Female	12 (75)
**Symptoms and signs, N (%)**	
Palpitations	8 (50)
Heat intolerance	6 (37.5)
Weight loss	6 (37.5)
Sweating	4 (25)
Tremors	7 (43.7)
Exophthalmos	4 (25)
Goiter	7 (43.7)
Headache	1 (6.2)
Visual field defect	3 (18.7)
**Diagnosis**	
GD and TSHoma co-existing	8 (50)
GD diagnosed first	4 (25)
TSHoma diagnosed first	4 (25)
TSH (Median [IQR], mIU/L)	1.95 (0.12–5.5)
FT3 (Median [IQR], pmol/L)	11.7 (7.6–19.7)
FT4 (Median [IQR], pmol/L)	47.6 (33.5–64.4)
**Antibodies positive, N (%)**	
TSH receptor Ab (N = 13)	10 (76.9)
Thyroid stimulating Ab (N = 3)	2 (66.6)
Anti TPO Ab (N = 7)Anti Tg Ab Ab (N = 7)	4 (57.1)
	4 (57.1)
High SHBG (N = 3)	3 (100)
Alpha-TSH/TSH molar ratio (N = 4)	
< 1	1 (25)
> 1	3(75)
TSH suppression after octreotide (N = 4)	4 (100)
**Thyroid uptake scan (N = 8)**	
Diffusely increased uptake	7 (87.5)
Hypoactive nodule	1 (12.5)
Pituitary imaging (N = 13)	
Macroadenoma	11 (84.6)
Microadenoma	2 (15.4)
Mean (SD) size (cm)	1.4 +/- 0.67
TRH Stimulation testing (N = 8)	
Normal response	1 (12.5)
Abnormal response	7 (87.5)
Somatostatin analog prior to surgery (N = 16)	4 (25)
Anti-thyroid drug initiation leading to worsening (N = 11)	3 (27.3)
Pituitary surgery performed (N = 16)	12 (75)
Immunostaining positive for TSHoma (N = 10)	8 (80)
**Final outcome (N = 16)**	
On maintenance treatment for GD	3 (18.7)
On maintenance somatostatin analogue	1 (6.25)
On maintenance betablocker only	1 (6.25)
Complete symptoms resolution after pituitary surgery	4 (25)
Not mentioned	6 (37.5)

TSHoma: Thyroid secreting hormone secreting pituitary adenoma; GD: Graves’ disease; TSH: Thyroid stimulating hormone, FT3: Free T3; FT4: Free T4; TRH: Thyrotropin releasing hormone; SHBG: Sex hormone binding globulin; TPO: Thyroid peroxidase; Tg: Thyroglobulin; SD: Standard deviation; Ab: Antibody

### Baseline characteristics of the study population

The mean age at diagnosis was 41 ± 13.6 years. 12 (75%) patients were females, and 4 (25%) were males.

### Clinical features of the study population

Eight (50%) patients had palpitations, 7 (43.7%) had goiter and tremors, 6 (37.5%) had weight loss and heat intolerance, 4 (25%) had increased sweating and exophthalmos, 3 (18.7%) had visual field defect, and 1 (6.2%) had a headache at presentation. Eight (50%) patients had co-existing GD and TSHoma at presentation, and 4 (25%) patients had GD diagnosed at initial presentation, followed by the development of TSHoma. 4 (25%) had TSHoma at the initial diagnosis complicated by GD development. In patients diagnosed with GD at first presentation, the mean duration between the development of TSHoma subsequently was 34.75 +/- 16.2 months. Similarly, the mean duration between the development of TSHoma and GD in patients initially presenting with TSHoma was 23 +/- 18.4 months.

### Biochemical and radiological data of the included patients

The median (IQR) initial TSH of the study population was 1.95 (0.12–5.5) mIU/L, the median (IQR) free T3 was11.7 (7.6–19.7) pmol/L, and the median (IQR) free T4 level was 47.6 (33.5–64.4) pmol/L. 10 (76.9%) patients had positive TSH receptor antibody levels, 4 (57.1%) had positive anti-thyroglobulin antibodies, 4 (57.1%) had positive anti-thyroid peroxidase (TPO) antibodies, and 2 (66.6%) had positive thyroid stimulating immunoglobulin (TSI). Three patients had sex hormone binding globulin (SHBG) levels done, and they were raised in all of them. Alpha TSH/TSH ratio was reported in 4 patients, and it was high (> 1) in 3 (75%) patients. A test to assess TSH suppression after octreotide administration was performed in 4 patients and showed TSH suppression in all. Thyrotropin-releasing hormone (TRH) stimulation was reported in eight patients, with abnormal results in 7(87.5%) patients. Of 8 patients with thyroid uptake scans done, diffuse uptake was noted in 7 (87.5%). The mean size of the pituitary tumor was 1.4 ± 0.67 cm. 11 (84.6%) out of 13 cases with reported pituitary tumor size had macroadenoma, and 2 (15.4%) had microadenoma.

### Treatment of patients with TSHoma and GD

Pituitary surgery was performed in 12 (75%) patients. 10 patients had immunostaining for thyrotrophs performed, and it was positive in 8 (80%) patients. At the last follow-up, 4 (25%) patients had complete resolution of symptoms after pituitary surgery, 3 (18.7%) were on maintenance treatment for GD, 1 (6.25%) on beta-blockers and 1 (6.25%) on somatostatin analog.

### Subgroup analysis of patients with concomitant TSHoma and GD

Table 3 summarizes the characteristics of patients with simultaneous TSHoma and GD at initial presentation. The mean (SD) age at diagnosis was 47.25 +/- 8.6 years. 6 (75%) patients were female. Palpitation, heat intolerance, tremors, and exophthalmos were the most common symptoms (50%), followed by visual field defect (25%) and goiter (25%). Mean (SD) TSH was 4.3 +/- 3.3 mIU/L, median (IQR) free T3 was 19.9 (6.6–19.7) pmol/Land mean (SD) free T4 was 45.5 +/- 13.6 pmol/L. TSH receptor antibody was positive in 6 (85.7%) patients. 7 (87.5%) had macroadenoma and 1 (12.5%) had microadenoma. The mean size of the pituitary tumor was 1.6 +/- 0.8 cm. 2 (25%) patients had complete resolution of symptoms after pituitary surgery, 2 (25%) required maintenance treatment for GD, while 1 (12.5%) required maintenance somatostatin analog treatment.

**Table 3:** Demographic characteristics, clinical characteristics and outcomes of patients with simultaneous presentation with TSHoma and GD

Variable	Result
Number of patients	8
Age in years (Mean +/- SD)	47.25 +/- 8.6
**Gender, N (%)**	
Male	2 (25)
Female	6 (75)
**Symptoms and signs, N (%)**	
Palpitations	4 (50)
Heat intolerance	4 (50)
Weight loss	1 (12.5)
Sweating	1 (12.5)
Tremors	4 (50)
Exophthalmos	4 (50)
Goiter	2 (25)
Headache	1 (12.5)
Visual field defect	2 (25)
TSH (Mean +/- SD, mIU/L)	4.3 +/- 3.3
FT3 (Median [IQR], pmol/ml)	9.9 (6.6–19.7)
FT4 (Mean +/- SD, pmol/L)	45.5 +/- 13.6
**Antibodies, N (%)**	
TSH receptor Ab (N = 7)	6 (85.7)
Thyroid stimulation Ab (N = 1)	1 (100)
Anti TPO Ab (N = 4)	3 (75)
Anti TG Ab (N = 3)	2 (66.6)
High SHBG (N = 2)	2 (100)
Alpha TSH/TSH molar ratio (N = 2)	
> 1	2 (100)
TSH suppression after octreotide (N = 2)	2 (100)
Thyroid uptake scan (N = 5)	
diffusely increased uptake	4 (80)
Hypoactive nodule	1 (20)
Pituitary imaging (N = 8)	
Macroadenoma	7 (87.5)
Not mentioned	1 (12.5)
Mean (SD) size of the adenoma (cm)	1.6 +/- 0.8
TRH Stimulation testing (N = 5)	
Normal response	2 (40)
Abnormal response	3 (60)
Somatostatin analogue prior to surgery (N = 8)	3 (37.5)
Anti-thyroid drug initiation leading to worsening. N = 4	2 (50)
Pituitary surgery was performed.	7 (87.5)
Pituitary irradiation	1 (12.5)
Immunostaining positive for TSHoma (N = 6)	5 (83.3)
**Final outcome (N = 8)**	
On maintenance treatment for GD	2 (25)
On maintenance somatostatin analogue	1 (12.5)
Complete symptoms resolution after pituitary surgery	2 (25)
Has residual mass with normal TSH	1 (12.5)
Not mentioned	2 (25)

TSHoma: Thyroid secreting hormone secreting pituitary adenoma; GD: Graves’ disease; TSH: Thyroid stimulating hormone, FT3: Free T3; FT4: Free T4; TRH: Thyrotropin releasing hormone; SHBG: Sex hormone binding globulin; TPO: Thyroid peroxidase; Tg: Thyroglobulin; SD: Standard deviation; Ab: Antibody

## Discussion

In this systematic review of 16 patients, we highlighted the clinical characteristics and outcomes of patients who developed hyperthyroidism due to TSHoma and GD. The mean age at diagnosis was 41 +/- 13.6 years. A female predominance (75%) was observed. Palpitation (50%) was the most common symptom at presentation50% of patients presented with simultaneous GD and TSHoma, 25% initially had GD with subsequent development of TSHoma, and 25% had TSHoma with later diagnosis of GD. The mean (SD) size of the pituitary tumor was 1.4 ± 0.67 cm. 84.6% had pituitary macroadenoma on the MRI. 75% of patients underwent pituitary surgery, and 88.9% had positive immunostaining for thyrotrophs. On follow-up, 25% had complete resolution of symptoms.

The pathophysiological mechanism of co-existing TSHoma and GD development remains to be established. TSH has been shown to increase the expression of TSH receptors (TSHR) in cultured thyroid cells at the messenger RNA level [25]. This provides an increased opportunity for the development of autoimmunity against the upregulated TSHR. Moreover, TSH also downregulates Fas, ICAM-1 (Intercellular adhesion molecule-1), and MHC (major histocompatibility complex) class II in the thyroid cells [26, 27]. In patients whose TSHoma is complicated by GD after pituitary surgery, a decrease in TSH level after the surgical intervention leading to activation of autoimmune responses against the thyroid glands may be a possible mechanism in such patients [18]. However, further studies are required to establish the exact pathophysiological mechanism.

Diagnosing TSHoma as a cause of hyperthyroidism is challenging, especially in patients with co-existing GD. The diagnosis of TSHoma is usually suspected when normal or high TSH levels are noted in the presence of high thyroid hormone levels. However, the degree of TSH elevation might be lower in patients with co-existing or pre-existing GD. In a review of 535 TSHoma cases, the median TSH level at diagnosis was 5.16 mU/L [28]. In our study of patients with TSHoma and GD, the mean (SD) TSH level was 2.82 +/- 3.1 mIU/L. Thus, a high index of suspicion is required to detect a co-existing TSHoma in patients being worked up for hyperthyroidism. Patients with untreated GD have TSH levels less than 0.1 mIU/L [15]. TSH levels that are detectable in high thyroid hormone levels should raise the suspicion of a concomitant TSHoma. Certain biochemical tests can further aid in diagnosing TSHoma. High alpha subunit/TSH ratio, high TSH alpha-subunit, and absent or diminished response of TSH to TRH stimulation have reported sensitivities of 83%, 75%, and 71%, respectively [29]. TSH suppression in response to short acting somatostatin analogues can also help in diagnosing TSHoma and differentiating it from other causes of SITSH. A 44.46% suppression in TSH level at 24 h compared to baseline has a sensitivity of 95% and specificity of 93.75% in diagnosing TSHoma [30]. Only four patients in our review reported an alpha-subunit/TSH ratio, which was elevated in 3 (75%) patients. Moreover, 7 (87.5%) of 8 patients had an abnormal TSH response to TRH stimulation. However, the lack of availability of these tests is a significant limitation in many centers. Most cases of TSHoma have pituitary macroadenoma on imaging. 2 systematic reviews on patients with TSHoma reported the prevalence of macroadenoma in up to 79% of patients with TSHoma [28, 31]. 84.6% of patients in our systematic review had pituitary macroadenoma as the etiology of TSH hypersecretion.

Management of patients with TSHoma and GD is challenging. Anti-thyroid drugs are a cornerstone of treatment in GD. TSHoma, on the other hand, is managed primarily with surgical removal of pituitary adenoma. Pre-operative management of these patients is vital as perioperative uncontrolled hyperthyroidism can lead to atrial fibrillation [32]. Pre-operative somatostatin analogues can lead to up to 73% reduction in TSH levels prior to surgery [33]. Post-operative remission has been noted in up to 70% of cases [34]. However, all the patients need close biochemical and imaging follow-up as recurrence rates ranging from 3 to 50% have been reported, particularly in the early years after the surgery [35]. If medical management is required either pre-operatively to decrease TSH levels and relieve hyperthyroidism symptoms before the surgical intervention or postoperatively in patients not completely cured with surgical intervention, then somatostatin analogs are the treatment of choice. If the diagnosis of TSHoma is overlooked in a patient with hyperthyroidism, there is a potential for an increase in adenoma size and worsening of symptoms with the institution of anti-thyroid drugs. Our systematic review reveals that of 8 patients with simultaneous TSHoma and GD, anti-thyroid drugs were initiated at initial presentation in 5 patients, of which one patient developed worsening symptoms of hyperthyroidism and one had a rise in TSH level. Similarly, in 4 patients who had TSHoma diagnosed initially, two were started on anti-thyroid drugs, of which 1 had an increase in TSH levels. This could be explained by the pathophysiological mechanism of the anti-thyroid drugs, which lead to a decrease in circulating thyroid hormone levels. A decrease in thyroid hormone levels leads to a decrease in negative feedback effect on the TSH secretion from the anterior pituitary. As a result, TSH levels rise, thus worsening clinical symptoms and increasing adenoma growth in patients with TSHoma [36]. Similarly, a Nelson syndrome like increase in invasive pituitary adenoma growth can occur in patients who inappropriately undergo radioactive iodine ablation or thyroidectomy to manage thyrotoxic symptoms in patients with TSHoma [36, 37].

To our knowledge, this is the first systematic review highlighting clinical characteristics and outcomes of patients with hyperthyroidism due to TSHoma and GD. It provides insight into patients’ diagnostic and management strategies with this clinically meaningful association. Our study has a few limitations. In our patient’s case, the IHC staining for TSH was negative in the resected pituitary adenoma tissue, and further stains including Pit-1 and GATA-2 were not available at our center. However, there was biochemical evidence of TSHoma in the patient and hence, he was managed as such. The major limitation of this systematic review is the inclusion of only small observational studies, i.e., case reports and case series. More extensive studies are needed to further improve the clinical knowledge gap regarding managing patients with this rare association.

## Conclusion

In a patient with hyperthyroidism, it is essential to recognize TSHoma as a possible etiology. Both TSHoma and GD can co-exist, and it is essential to identify this rare association as it can significantly impact treatment strategies. Initiation of anti-thyroid drugs in a patient with co-existing TSHoma and GD can worsen the symptoms and increase the size of the pituitary adenoma. Non-suppressed TSH level, abnormal response to TRH stimulation, and a high alpha-subunit/TSH molar ratio are some biochemical tests that can aid in identifying concomitant TSHoma in a patient with hyperthyroidism who is being worked up for GD.

## Data Availability

Data sharing is not applicable.
